# Clinical impact of physician staffing transition in intensive care units: a retrospective observational study

**DOI:** 10.1186/s12871-022-01905-0

**Published:** 2022-11-26

**Authors:** Yosuke Fujii, Kiichi Hirota, Kentaro Muranishi, Yumiko Mori, Kei Kambara, Yoshitaka Nishikawa, Mitsuko Hashiguchi

**Affiliations:** 1 Department of Anesthesia, Otsu City Hospital, 2-9-9 Motomiya, Otsu, Shiga 520-0804 Japan; 2 Department of Human Stress Response Science, Institute of Biomedical Science, Kansai Medical University, 2-3-1 Shin-machi, Hirakata, Osaka 573-1191 Japan; 3 Department of Emergency and Intensive Care Medicine, Otsu City Hospital, 2-9-9 Motomiya, Otsu, Shiga 520-0804 Japan; 4 Department of Health Informatics, Kyoto University School of Public Health, Yoshida Konoe-cho, Sakyo-ku, Kyoto, Kyoto 606-8501 Japan

**Keywords:** Intensive care unit, ICU staffing, High-intensity staffing, Low-intensity staffing, Intensivist, Interrupted time-series analysis

## Abstract

**Background:**

Intensivists play an essential role in improving the outcomes of critically ill patients in intensive care units (ICUs). The transition of ICU physician staffing from low-intensity ICUs (elective intensivist or no intensivist consultation) to high-intensity ICUs (mandatory intensivist consultation or a closed ICU) improves clinical outcomes. However, whether a transition from high-intensity to low-intensity ICU staffing affects ICU outcomes and quality of care remains unknown.

**Methods:**

A retrospective observational study was conducted to examine the impact of high- versus low-intensity staffing models on all-cause mortality in a suburban secondary community hospital with 400 general beds and 8 ICU beds. The ICU was switched from a high-intensity staffing model (high-former period) to low-intensity staffing in July 2019 (low-mid period) and then back to high-intensity staffing in March 2020 (high-latter period). Patients admitted from the emergency department, general ward, or operating room after emergency surgery were enrolled in these three periods and compared, balancing the predicted mortality and covariates of the patients. The primary outcome was all-cause mortality analyzed using hazard ratios (HRs) from Cox proportional hazards regression. An interrupted time-series analysis (ITSA) was also conducted to evaluate the effects of events (level change) and time.

**Results:**

There were 962 eligible admissions, of which 251, 213, and 498 occurred in the high-former, low-mid, and high-latter periods, respectively. In the matched group (*n* = 600), the all-cause mortality rate comparing the high-former period with the low-mid period showed an HR of 0.88 [95% confidence interval (CI), 0.56, 1.39; *p* = 0.58] and that comparing the high-latter period with the low-mid period showed an HR of 0.84 [95% CI, 0.54, 1.30; *p* = 0.43]. The result for comparison between the three periods was *p* = 0.80. ITSA showed level changes of 4.05% [95% CI, -13.1, 21.2; *p* = 0.63] when ICU staffing changed from the high-former to the low-mid period and 1.35% [95% CI, -13.8, 16.5; *p* = 0.86] when ICU staffing changed from the low-mid to the high-latter period.

**Conclusion:**

There was no statistically significant difference in all-cause mortality among the three ICU staffing periods. This study suggests that low-intensity ICU staffing might not worsen clinical outcomes in the ICU in a medium-sized community hospital. Multiple factors, including the presence of an intensivist, other medical staff, and practical guidelines, influence the prognosis of critically ill patients.

**Supplementary Information:**

The online version contains supplementary material available at 10.1186/s12871-022-01905-0.

## Introduction

Intensivists play an essential role in managing intensive care units (ICUs) and delivering high-quality intensive care. High-intensity intensivist staffing improves the quality of critical care and contributes to better clinical outcomes in terms of mortality, duration of mechanical ventilation, and length of ICU stay (LOS) [1, 2]. According to the Society of Critical Care Medicine's guidelines for ICU admission, discharge, and triage in the United States [3] and the Leapfrog standards for critical care [4], ICUs should be staffed by intensivists who can coordinate and manage the care of critically ill patients. Further, ICU facility standards require the presence of a doctor who primarily works in the ICU.

Although the crucial functions of intensivists are widely recognized, there are not sufficient qualified intensivists to staff all ICUs in Japan [5]. At Otsu City Hospital, ICU physicians had comprised board-certified intensivist(s) for more than a decade; however, there was a temporary change in ICU physician staffing over the past few years. It was unclear whether the prognosis and quality of intensive care would decrease or remain unchanged once intensivists were absent in ICU management. It is impossible to directly compare the two situations of ICU management with and without intensivists using randomized controlled trials because random assignment of patients to the care of either intensivists or in-hospital physicians from other specialties is neither practical nor ethical [2]. To overcome this difficulty, our study combined matching to balance covariates [6] and an interrupted time-series analysis (ITSA) [7] to evaluate the clinical effect of transitioning between high-intensity and low-intensity staffing on mortality.

## Methods

### Ethical approval and consent to participate

This study was approved by the ethics committee of Otsu City Hospital (approval number 23; approval date, June 18, 2020). This study was conducted under the Declaration of Helsinki as a statement of ethical principles for medical research developed by the World Medical Association and STROBE reporting guidelines. The need for informed consent was waived by the ethics committee of Otsu City Hospital due to the retrospective nature of the study.

### Data

The patients were admitted to the ICU from the emergency department, general ward, or operating room after emergency surgery. Exclusion criteria were patients under 16 years of age and patients with missing data. The study period was from November 1, 2018 to September 30, 2021. Otsu City Hospital, which has approximately 400 available beds and 8 ICU beds (6 ICU beds until October 2018), is a secondary hospital that offers emergency care to patients who may require hospitalization and provides intensive care for cases of acute coronary syndrome, stroke, and sepsis. It serves a population of approximately 340,000 people and manages 12,000 emergency room visits and 4,000 emergency transports every year. The study design considered the before–after setting because it investigated a policy change or transition at a given time point [1–3, 8–10]. Three periods were defined for comparison: 1) high-former, which indicated high-intensity ICU staffing with full-time board-certified intensivist(s) from November 1, 2018 to June 30, 2019; 2) low-mid, which indicated low-intensity ICU staffing without any full-time board-certified intensivists from July 1, 2019 to March 31, 2020; and 3) high-latter, which indicated high-intensity ICU staffing with full-time board-certified intensivist(s) from April 1, 2020 to September 30, 2021. Seven or eight physicians staffed the ICU department in the high-former period; two or three physicians, including intensivists, exclusively staffed the ICU and managed a high-intensity ICU, which included mandatory intensivist consultation [1], or a closed ICU. Two intensivists were staffed until January 2019, after which one intensivist was staffed. Physicians dedicated to the ICU were the responsible physicians for ICU patients during the day-time. During the nighttime, one of them or full-time in-house specialists in other fields were on duty. In the low-mid period, two physicians who were board-certified specialists in other fields managed the ICU along with full-time or part-time doctors, and the ICU continued to offer 24/7 care. During this period, there were no full-time intensivists available, and thus the unit functioned as an open ICU, so this term was categorized as low-intensity ICU staffing [1]. A new intensivist arrived on April 1, 2020 and became a new member of the ICU. Four physicians, including newly hired intensivists with several part-time intensivists, restarted high-intensity ICU staffing; this period was defined as high-latter (Table 1).

**Table 1:** The number of full-time physicians during the three research periods

Period	High-former Nov 2018–Jun 2019	Low-mid Jul 2019–Mar 2020	High-latter Apr 2020–Sep 2021
Intensivists	2 (–Jan 2019)	0	1 (–Mar 2021)
	1 (Feb 2019–)		3 (Apr 2021–)
Fellows	2	2	3 (–Mar 2021)
			1 (Apr 2021–)
Senior residents	4	0	0

### Statistical analyses

Matching based on the Acute Physiology and Chronic Health Evaluation (APACHE) II score was used to balance covariates prior to survival analysis. APACHE II and its related clinical values were collected from electronic health records within the first 24 h of ICU admission [11]. The collected values and data to be analyzed included age, sex, white blood cell count (10^3^/mm^3^), hematocrit (%), Na^+^ level (mmol/L), K^+^ level (mmol/L), creatinine level (mg/dL), pH, partial pressure of arterial oxygen (mmHg), mean arterial pressure (mmHg), heart rate (beats per minute), respiratory rate (breaths per minute), body temperature (℃), and Glasgow Coma Scale score. To convert the APACHE II score to quantitative prognostic probability, additional information was collected, such as the requirement for emergency surgery, the reasons for ICU admission, and APACHE II diagnostic category weight according to the principal reason for ICU admission for calculating the predicted mortality (Supplementary information). Cases with no missing data were eligible for the analysis. The conventional paired matching was no longer applicable because there were multiple treatment groups [12–14]. The low-mid period was determined as the reference group, and the high-former vs. low-mid matching was done first. Then, matched samples in the low-mid (reference) were extracted and fixed. Next, the high-latter vs. low-mid matching was conducted. The samples in the high-former and high-latter periods matched with the same sample in the low-mid period were combined into a trio. Matchings were performed at a 1:1 ratio using 0.2 calipers without replacement to minimize the number of biased variables using the *Match* function in the Matching package in R [15].

ITSA was conducted to consider time trends [7]. An original ITSA focused on a segmented regression, presented as:

$${Y}_{t}={\beta }_{0}+{\beta }_{1}t+{\beta }_{2}I\left(t\right)+{\beta }_{3}T\left(t\right)+{\epsilon }_{t},$$

where ${Y}_{t}$ is the outcome at a given time point $t$, $$I\left(t\right)=\left\{\begin{array}{cc}0& \textrm{if}\ t\le {t}_{0}\\ 1& \textrm{otherwise}\end{array}\right.$$ is the event variable, $$T\left(t\right)=\left\{\begin{array}{cc}0& \textrm{if}\ t\le {t}_{0}\\ t-{t}_{0}& \textrm{otherwise}\end{array}\right.$$ is the indicator of elapsed time from the event, ${t}_{0}$ is the time of the event, ${\epsilon }_{t}$ is an error term, and $\beta$ s are regression coefficients. A segmented regression estimates ${\beta }_{2}$ (step or level change) and ${\beta }_{3}$ (slope or trend change) to evaluate the impact of the event. Schaffer et al. recommended the adaptation of the autoregressive integrated moving average (ARIMA) model to ITSA because the linear regression model assumed that the error terms were independent and not correlated between each data point, but longitudinal time series data typically exhibited features of non-stationarity, autocorrelation, and seasonality [16]. The set of parameters of the ARIMA model was determined by the *auto.arima* function in the forecast package in R [17] based on minimizing the Akaike or Bayesian information criterion [16]. The interest of analysis was the 30-day survival rate of covariate-balanced patients who were admitted to the ICU grouped by month, and the unit of time point for ITSA was months. Regression was performed using the *lm_robust* function in the estimatr package in R [18], accounting for heteroscedasticity based on the HC3 heteroscedasticity consistent covariance matrix [19, 20]. Heteroscedasticity was tested by the Breusch-Pagen test.

The primary outcome was all-cause mortality from the day of ICU admission. The secondary outcomes were the numbers of ICU and hospital deaths, the lengths of ICU and hospital stays, all-cause mortality stratified by APACHE II score-based predicted mortality according to the severity with a threshold of ≤ 30%, and level and trend changes estimated via ITSA. All-cause mortality was analyzed using the Cox proportional hazards model. Continuous data were expressed as the mean ± standard deviation, and categorical data were expressed as numbers (percentages). The analysis of variance for unmatched samples, and Friedman test for matched samples were used for continuous values and chi-squared test was used for categorical data. A *p*-value of < 0.05 was considered significant. Probabilistic uncertainty was represented using confidence intervals (CIs). Statistical analyses were performed using the R programming language and software (version 3.6.2).

## Results

### Baseline characteristics and matching

**Fig. 1:**
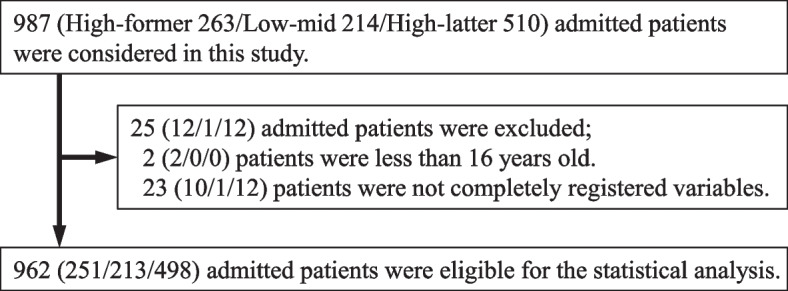
The number of eligible patients in this study

This study considered 987 admitted patients, among which 263 were enrolled in the high-former period, 214 were enrolled in the low-mid period, and 510 were enrolled in the high-latter period. Of these, 251, 213, and 498 patients in the high-former, low-mid, and high-latter periods, respectively, had complete data and were eligible for the outcome analysis (Fig. 1). The patients' mean age was 72.1 ± 16.1 years, 36.4% were > 80 years of age, and 57.5% were men. Patients admitted in the low-mid period had higher APACHE II scores and predicted mortality. The matching procedure matched 600 patients. Table 2 shows the baseline characteristics of the admissions and the parameters associated with the APACHE II-predicted mortality among the three periods before and after matching. All data were adequately balanced after matching.

**Table 2:** Patient characteristics. Categorical data are expressed as numbers with percentages (%) and were tested using the chi-squared test. Mean and standard deviation (SD) are provided for quantitative variables, and variables were tested using the analysis of variance for before-matching data and the Friedman test for after-matching data. APACHE, Acute Physiology and Chronic Health Evaluation; ER, emergency room; GCS, Glasgow Coma Scale; ICU, intensive care unit; PaO_2_, partial pressure of arterial oxygen; WBC, white blood cell

	Before matching				After matching			
Period	High-former	Low-mid	High-latter	*p-*value	High-former	Low-mid	High-latter	*p-*value
n	251	213	498		200	200	200	
Predicted mortality (%)	26.1 (22.4)	31.7 (23.5)	30.6 (22.5)	0.012	30.0 (22.6)	30.0 (22.6)	30.0 (22.7)	0.577
APACHE II score	15.3 (8.09)	17.7 (7.64)	17.5 (7.32)	< 0.001	16.9 (7.83)	17.2 (7.47)	17.5 (7.23)	0.863
Age (years)	73.3 (15.9)	74.3 (14.5)	70.5 (16.7)	0.006	75.2 (14.5)	74.3 (14.3)	71.8 (15.4)	0.127
Mean arterial pressure (mmHg)	88.9 (35.2)	92.7 (39.6)	89.2 (42.5)	0.511	87.7 (38.3)	93.6 (40.1)	88.2 (43.4)	0.059
Heart rate (beats/min)	81.1 (23.1)	83.4 (27.4)	81.4 (26.5)	0.562	81.7 (24.0)	83.2 (27.2)	82.5 (26.4)	0.870
Respiratory rate (breaths/min)	18.7 (7.71)	20.7 (9.01)	20.7 (8.87)	0.006	18.9 (8.12)	20.5 (8.92)	21.3 (8.93)	0.095
Temperature (℃)	36.9 (0.99)	37.1 (0.93)	37.1 (0.95)	0.052	36.9 (1.06)	37.0 (0.94)	37.1 (0.97)	0.371
GCS score	12.0 (3.94)	11.4 (4.30)	11.1 (4.59)	0.028	11.5 (4.10)	11.64 (4.13)	11.4 (4.44)	0.861
Sodium (mmol/L)	137.8 (4.87)	139.6 (5.66)	138.7 (5.17)	< 0.001	137.9 (5.14)	139.6 (5.57)	138.9 (5.59)	0.052
Potassium (mmol/L)	3.81 (0.59)	3.77 (0.58)	3.80 (0.59)	0.739	3.82 (0.61)	3.77 (0.59)	3.74 (0.53)	0.541
pH	7.42 (0.08)	7.42 (0.07)	7.41 (0.08)	0.084	7.42 (0.08)	7.42 (0.07)	7.41 (0.08)	0.357
PaO_2_ (mmHg)	83.7 (23.0)	81.2 (21.9)	83.4 (17.7)	0.344	81.3 (22.0)	81.1 (22.25)	83.4 (18.4)	0.114
WBC count (10^3^/mm^3^)	10.1 (5.08)	10.0 (5.46)	10.7 (7.16)	0.318	10.4 (5.21)	9.43 (4.53)	10.70 (6.40)	0.349
Creatinine (mg/dL)	1.21 (1.25)	1.48 (1.93)	1.46 (2.29)	0.205	1.27 (1.25)	1.51 (1.98)	1.55 (3.08)	0.732
Hematocrit (%)	33.5 (6.21)	32.7 (6.34)	33.0 (6.27)	0.353	33.1 (6.20)	32.9 (6.28)	32.7 (6.37)	0.633
APACHE II diagnostic category weight	-1.43 (1.51)	-0.96 (1.30)	-1.07 (1.33)	< 0.001	-1.07 (1.27)	-1.05 (1.26)	-1.05 (1.26)	0.130
Sex				0.895				0.826
Female	106 (42.2)	88 (41.3)	215 (43.2)		87 (43.5)	81 (40.5)	85 (42.5)	
Male	145 (57.8)	125 (58.7)	283 (56.8)		113 (56.5)	119 (59.5)	115 (57.5)	
The reason for ICU admission				0.818				0.630
Emergency surgery	50 (19.9)	45 (21.1)	95 (19.1)		40 (20.0)	41 (20.5)	34 (17.0)	
ER or ward	201 (80.1)	168 (78.9)	403 (80.9)		160 (80.0)	159 (79.5)	166 (83.0)	
Severe organ failure				0.854				0.330
None	200 (79.7)	174 (81.7)	403 (80.9)		153 (76.5)	165 (82.5)	158 (79.0)	
Severe organ failure	51 (20.3)	39 (18.3)	95 (19.1)		47 (23.5)	35 (17.5)	42 (21.0)	

### Primary outcomes

**Fig. 2:**
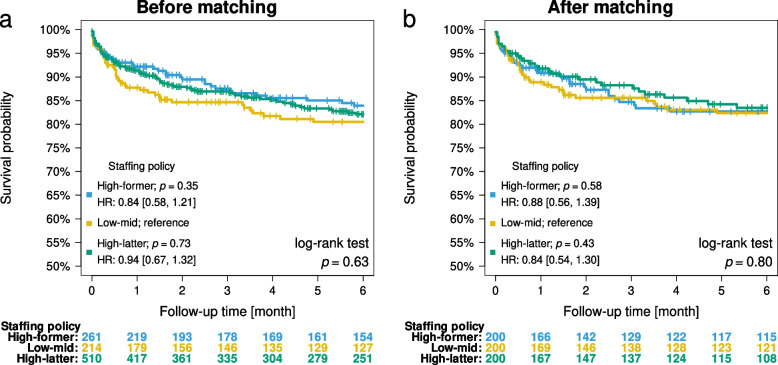
Patients' survival rates in each intensive care unit staffing period before matching (**a**) and after matching (**b**). HR, hazard ratio

The Cox proportional hazard model of the comparison between the high-former and low-mid periods showed a hazard ratio (HR) of 0.84 [95% CI, 0.58, 1.21; *p* = 0.35], and that between the high-latter and low-mid periods showed an HR of 0.94 [95% CI, 0.67, 1.32; *p* = 0.73]. The result of the comparison of the primary outcome between the three periods before matching was *p* = 0.63 (Fig. 2a). Among the matched groups, the result of the comparison between the high-former and low-mid periods showed an HR of 0.88 [95% CI, 0.56, 1.39; *p* = 0.58], and that between the high-latter and low-mid periods showed an HR of 0.84 [95% CI, 0.54, 1.30; *p* = 0.43]. The result of the comparison of the primary outcome among the three periods was *p* = 0.80 (Fig. 2b).

### Secondary outcomes

The numbers of ICU deaths among the high-former, low-mid, and high-latter periods were 12 (4.78%), 10 (4.69%), and 18 (3.61%) (*p* = 0.68) before matching and 10 (5.00%), 9 (4.50%), and 5 (2.50%) (*p* = 0.40) after matching. The numbers of hospital deaths among the high-former, low-mid, and high-latter periods were 54 (21.5%), 40 (18.8%), and 89 (17.9%) (*p* = 0.49) before matching and 49 (24.5%), 35 (17.5%), and 35 (17.5%) (*p* = 0.13) after matching (Table 3). The LOSs among the high-former, low-mid, and high-latter periods were 3.18 days, 3.88 days, and 3.52 days (*p* = 0.09) before matching and 3.47 days, 3.88 days, and 3.30 days (*p* = 0.17) after matching. The lengths of hospital stay among the high-former, low-mid, and high-latter periods were 30.5 days, 34.0 days, and 32.2 days (*p* = 0.51) before matching and 32.7 days, 33.7 days, and 32.8 days (*p* = 0.39) after matching (Table 3).

**Table 3:** The numbers of deaths and lengths of stay in the ICU and the hospital. Categorical data are expressed as numbers with percentages (%) and were tested using the chi-squared test. Mean and interquartile ranges are provided for the length of stay, and they were tested using the analysis of variance for before-matching data and the Friedman test for after-matching data. ICU, intensive care unit; IQR, interquartile range

	Before matching				After matching			
Period	High-former	Low-mid	High-latter	*p-*value	High-former	Low-mid	High-latter	*p-*value
n	251	213	498		200	200	200	
Death (%)								
ICU	12 (4.78)	10 (4.69)	18 (3.61)	0.68	10 (5.00)	9 (4.50)	5 (2.50)	0.40
Hospital	54 (21.5)	40 (18.8)	89 (17.9)	0.49	49 (24.5)	35 (17.5)	35 (17.5)	0.13
Length of stay [IQR]								
ICU	3.18 [1, 4]	3.88 [1, 5]	3.52 [1, 4]	0.09	3.47 [1, 4]	3.88 [1, 5]	3.30 [1, 4]	0.17
Hospital	30.5 [12, 44]	34.0 [12, 46]	32.2 [11, 41]	0.51	32.7 [13, 47]	33.7 [13, 45]	32.8 [12, 42]	0.39

**Fig. 3:**
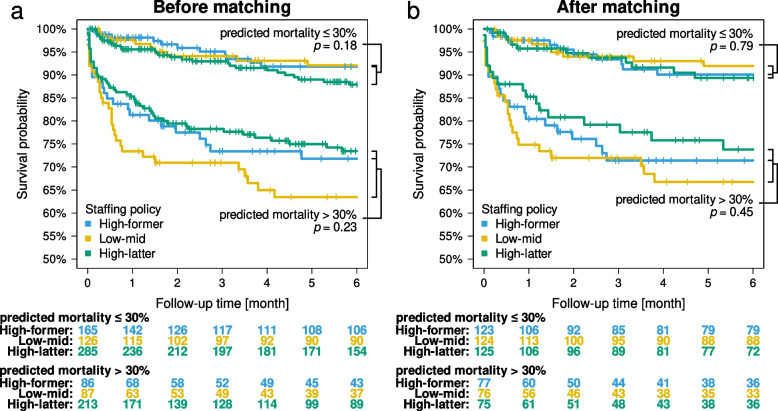
The survival rate stratified by the severity of predicted mortality according to Acute Physiology and Chronic Health Evaluation (APACHE) II scores. Patients are divided into non-severe (predicted mortality ≤ 30%) and severe (predicted mortality > 30%) cases. Survival curves are drawn for data (**a**) before matching and (**b**) after matching

For the secondary outcomes of the subpopulation stratified by APACHE II score-based predicted mortality according to the severity with a threshold of ≤ 30%, the all-cause mortality rates of less severe patients showed an HR of 0.95 [95% CI, 0.51, 1.77; *p* = 0.86] between the high-former and low-mid periods and an HR of 1.51 [95% CI, 0.85, 2.71; *p* = 0.16] between the high-latter and low-mid periods. The all-cause mortality rates of severe patients showed an HR of 0.79 [95% CI, 0.49, 1.28; *p* = 0.34] between the high-former and low-mid periods and an HR of 0.69 [95% CI, 0.46, 1.05; *p* = 0.09] between the high-latter and low-mid periods (Table 4). The comparison among the three periods showed *p* = 0.18 for less severe patients and *p* = 0.23 for severe patients before matching (Fig. 3a). After matching, the all-cause mortality rates of less severe patients showed an HR of 1.22 [95% CI, 0.65, 2.30; *p* = 0.53] between the high-former and low-mid periods and an HR of 1.22 [95% CI, 0.60, 2.45; *p* = 0.58] between the high-latter and low-mid periods. The all-cause mortality rates of severe patients showed an HR of 0.87 [95% CI, 0.52, 1.45; *p* = 0.60] between the high-former and low-mid periods and an HR of 0.70 [95% CI, 0.39, 1.23; *p* = 0.21] between the high-latter and low-mid periods (Table 4). The comparison among the three periods showed *p* = 0.79 for less severe patients and *p* = 0.45 for severe patients (Fig. 3b).

**Table 4:** Results of secondary outcomes stratified by predicted mortality. All-cause mortality of the low-mid period is compared to those of the high-former and high-latter periods. HR, hazard ratio; CI, confidence interval

	Before matching		After matching	
Comparison	HR [95% CI]	*p-*value	HR [95% CI]	*p-*value
Non-severe (predicted morality $\le$ 30)				
High-former	0.95 [0.51, 1.77]	0.86	1.22 [0.65, 2.30]	0.53
High-latter	1.51 [0.85, 2.71]	0.16	1.22 [0.60, 2.45]	0.58
Severe (predicted mortality > 30)				
High-former	0.79 [0.49, 1.28]	0.34	0.87 [0.52, 1.45]	0.60
High-latter	0.69 [0.46, 1.05]	0.09	0.70 [0.39, 1.23]	0.21

**Fig. 4:**
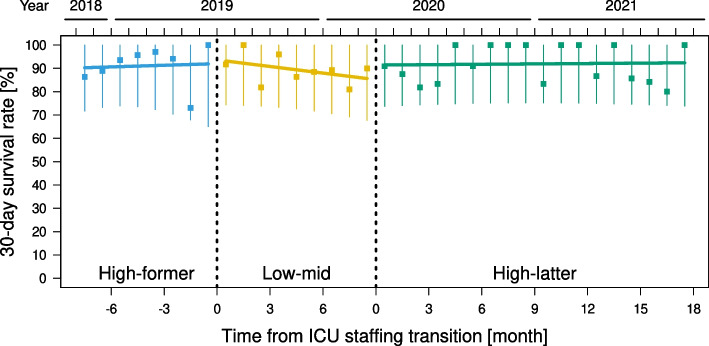
Interrupted time-series analysis (ITSA) of the effect of the transition of ICU staffing. Thirty-day survival rates (square dots) with 95% prediction intervals (vertical error bars) are shown. At the transition from the high-former period to the low-mid period, ITSA shows a level change of 4.05% [95% CI, -13.1, 21.2; *p* = 0.63] and a trend change of -0.94%/month [95% CI, -2.80, 0.92; *p* = 0.31]. At the transition from the low-mid period to the high-latter period, ITSA shows a level change of 1.35% [95% CI, -13.8, 16.5; *p* = 0.86] and a trend change of 0.05%/month [95% CI, -0.78, 0.89; *p* = 0.89] for the 30-day survival rate. ITSA, interrupted time-series analysis; ICU, intensive care unit; CI, confidence interval

The ARIMA model was determined as $\left(p, d, q\right)=(0, 0, 0),$ indicating that the autocorrelation order of the model (*p*), the moving average order of the model (*q*), and the time series difference (*d*) were 0, and no seasonality was appropriately fitted. The result of the Breusch-Pagan test for heteroscedasticity was *p* = 0.08. At the transition from the high-former period to the low-mid period, ITSA showed a level change of 4.05% [95% CI, -13.1, 21.2; *p* = 0.63] and a trend change of -0.94%/month [95% CI, -2.80, 0.92; *p* = 0.31]. At the transition from the low-mid period to the high-latter period, ITSA showed a level change of 1.35% [95% CI, -13.8, 16.5; *p* = 0.86] and a trend change of 0.05%/month [95% CI, -0.78, 0.89; *p* = 0.89] (Fig. 4 and Table 5).

**Table 5:** The results of the interrupted time-series analysis. CI, confidence interval

Period	Level (%) [95% CI]	*p-*value	Trend (%/month) [95% CI]	*p-*value
High-former	90.0 [77.2, 100.0]	< 0.001	0.23 [-3.87, 4.34]	0.91
Low-mid	4.05 [-13.1, 21.1]	0.63	-0.94 [-2.80, 0.92]	0.31
High-latter	1.35 [-13.8, 16.5]	0.86	0.05 [-0.78, 0.89]	0.89

## Discussion

When ICU staffing transitions from low intensity to high intensity, there are significant improvements in outcomes [1, 2] in medical [21], surgical [10, 22], and specialized (cardiovascular [9, 23, 24], neurological [25–27], and pediatric [28]) ICUs. The Leapfrog Group's ICU Physician Staffing Safety Standards recommend high-intensity staffing [4] based on the results of prior studies [21–23, 29]. Despite such compelling evidence, cost barriers or a lack of available expertise prevents hospitals from employing full-time intensivists [30]; only 47% of hospitals in the United States met this standard in a 2015 survey [31]. Consequently, other specialists or hospitalists are forced to care for ICU patients [32]. Some studies have clarified that ICU management does not improve mortality rates [8, 9, 29, 32] and that its effectiveness is controversial, whereas some guidelines recommend placing intensivists in the ICU [3, 4]. Conversely, it is intuitively easy to imagine that an absence of intensivists may reduce ICU management quality. However, our study showed that the transitions from high-intensity to low-intensity ICU staffing and from low-intensity to high-intensity ICU staffing did not significantly change clinical outcomes for all-cause mortality. This might have occurred due to the following reasons. First, even during the low-mid period, at least one well-trained full-time or part-time physician from another field was staffed to enable continuous ICU staffing (the 24/7 staffing model). In addition to implementing the 24/7 staffing model of ICU physicians, multidisciplinary medical teams comprising nurses, pharmacologists, clinical engineers, physical therapists, and medical social workers contributed to ICU management throughout the study period. The remaining staff (i.e., the board-certified respiratory internist and the newly hired physician specializing in emergency medicine) consistently followed the ABCDEF bundle [33] – an evidence-based approach that targets critically ill patients [34] – during the low-mid period to optimize treatment for patients in the ICU by collaborating with multidisciplinary medical teams. Despite the downgrade of the staffing model, the increased adherence to clinical practice guidelines [2] and the presence of multidisciplinary teams in the ICU [35, 36] would have affected patient outcomes. Second, the impact of the presence of intensivists on mortality might not be as significant as expected in earlier studies [37]. Early studies that demonstrated a relationship between mortality reduction and intensivist staffing models were mostly from single-center and before–after analyses conducted in the 1990s or 2000s [1, 2]. Furthermore, ICU and hospital mortality rates varied by decade, decreasing in the 1980s and 2000s but not in the 1990s or 2010s [2]. Recent multicenter analyses showed no significant association between high-intensity ICU staffing and mortality [38, 39]. These studies suggest that the relationship between intensivist staffing and patient mortality is weaker than previously thought. Third, the transition in intensivist staffing probably has only a limited impact on mortality in small- and medium-volume hospitals. A closed ICU implementing a 24/7 ICU intensivist staffing model in an academic hospital reduced LOS and generated significant cost savings [40]. Furthermore, a recent study revealed that the presence of 24/7 in-house ICU intensivists positively affects the quality of care for critically ill patients in high-acuity, high-volume centers; however, the benefits could not be sufficiently extrapolated to low-acuity, low-volume hospitals to justify the increase in staffing needs and costs [41]. In our study, 24/7 staffing with trained physicians and multidisciplinary medical teams played an essential role in maintaining the quality of intensive care in a relatively small community hospital. Lastly, the statistical power was too small to detect any significant increase or decrease in mortality due to the small mortality effect size. A study that demonstrated an improvement in the 28-day mortality of oncology patients in an ICU implementing a high-intensity staffing model reduced the mortality rate from 47.69% to 29.84% [42]. Since oncology patients are more severely ill (median APACHE II score, 20) than the general ICU patient population, there is room to improve mortality rates. However, it is difficult to significantly reduce the mortality rate in such a low-mortality population [9]. Since our study's participants had lower ICU and hospital mortality in any period than those seen in the Japanese database (6.3% ICU mortality for critically ill adults [43]), it had low statistical power due to the small effect size.

The importance of employing intensivists in ICUs and healthcare systems is widely recognized [44]. Furthermore, the importance of employing full-time ICU physicians is highlighted in Japan [45, 46]. However, the implementation of high-quality ICU management remains inadequate, even in developed countries. In the United States, 53% of ICUs did not meet Leapfrog's standard according to a 2015 survey [31]. In Canada, 85% of ICUs could not satisfy the professional standards of practice recommended by the relevant guidelines, and 49% of them did not have dedicated in-house ICU physicians according to a 2006 survey [47]. Lastly, only 39% of ICUs in Japan employed board-certified intensivists according to a 2008 survey [36]. Intensivists are a rare resource, and the feasibility of broad-based expansion of ICU staffing will take time. Increased adherence to clinical practice guidelines [2] and the presence of multidisciplinary teams in the ICU [35, 36] would positively impact patient outcomes.

This study had some limitations. First, the APACHE II score-based matching analysis did not necessarily control group bias. Second, this study used a single-center, before–after setting. Since before–after analyses suffer from secular trends [2, 8], our results cannot be simply generalized and extrapolated to other settings. Third, due to the unavailability of data, we did not account for other clinical outcomes that could have realized intensivists' maximum potential, such as the duration of mechanical ventilation, the optimization of sedation, and cost-effective management. A significant difference might have occurred in these surrogate clinical indicators.

## Conclusions

All-cause mortality did not significantly change with the transition from 24/7 high-intensity ICU staffing with board-certified intensivist(s) to 24/7 low-intensity ICU staffing without the board-certified staff and then back to high-intensity ICU staffing. Our results indicated that the existence of intensivists is not the sole factor influencing clinical outcomes in the ICU and that intensivists play a vital role in intensivist-led ICU rounds to treat critically ill patients by collaborating with intra-hospital physicians and multidisciplinary medical teams.

## Supplementary Information

**Additional file 1.**

## Data Availability

The datasets used and analyzed during the current study are available from the corresponding author upon reasonable request.

## Abbreviations

ICU: Intensive care unit
APACHE: Acute Physiology and Chronic Health Evaluation
LOS: Length of ICU stay
ITSA: Interrupted time-series analysis
ARIMA: Autoregressive integrated moving average
CI: Confidence interval
HR: Hazard ratio
IQR: Interquartile range
