# Supplementary material for: Clinical impact of physician staffing transition in intensive care units: a retrospective observational study
Source: BMC Anesthesiol. 2022 Nov 26;22:362. doi: 10.1186/s12871-022-01905-0 (PMC9701368; doi:10.1186/s12871-022-01905-0)
Supplement: Supplementary file 1 — Additional file 1. [file 12871_2022_1905_MOESM1_ESM.docx]

To compute the predicted mortality for a group of acutely ill patients, the risk $f(x)$ hospital death was computed for each individual using the following equation:

$$f\left( x \right)=\frac{1}{1+e^{-x}},$$

$$x=-3.517+0.146A+0.603I+D$$

where $A$ is Acute Physiological and Chronic Health Evaluation (APACHE) II score, $I$ is the indicator if the patient was admitted after emergency surgery $I=1$ or other $I=0$, and $D$ is diagnostic category weight or coefficient as shown in the supplemental Table S1 [1].

| Nonoperative patients | |
| --- | --- |
| Asthma/allergy | -2.108 |
| Chronic obstructive pulmonary disease | -0.367 |
| Pulmonary edema (noncardiogenic) | -0.251 |
| Postrespiratory arrest | -0.168 |
| Aspiration/poisoning/toxic | -0.142 |
| Pulmonary embolus | -0.128 |
| Infection | 0 |
| Neoplasm | 0.891 |
| Hypertension | -1.798 |
| Rhythm disturbance | -1.368 |
| Congestive heart failure | -0.424 |
| Hemorrhagic shock/hypovolemia | 0.493 |
| Coronary artery disease | -0.191 |
| Sepsis | 0.113 |
| Postcardiac arrest | 0.393 |
| Cardiogenic shock | -0.259 |
| Dissecting thoracic/abdominal aneurysm | 0.731 |
| Multiple trauma | -1.228 |
| Head trauma | -0.517 |
| Seizure disorder | -0.584 |
| ICH/SDH/SAH | 0.723 |
| Drug overdose | -3.353 |
| Diabetic ketoacidosis | -1.507 |
| GI bleeding | 0.334 |
| If not in one of the specific groups above, then which major vital organ system was the principal reason for admission? | |
| Metabolic/renal | -0.885 |
| Respiratory | -0.980 |
| Neurologic | -0.759 |
| Cardiovascular failure or insufficiency from | 0.470 |
| Gastrointestinal | 0.501 |
| Postoperative patients | |
| Multiple trauma | -1.684 |
| Chronic cardiovascular disease | -1.376 |
| Peripheral vascular surgery | -1.315 |
| Heart valve surgery | -1.261 |
| Craniotomy for neoplasm | -1.245 |
| Renal surgery for neoplasm | -1.204 |
| Renal transplant | -1.042 |
| Head trauma | -0.955 |
| Thoracic surgery for neoplasm | -0.802 |
| Craniotomy for ICH/SDH/SAH | -0.788 |
| Laminectomy and other spinal cord surgery | -0.699 |
| Hemorrhagic shock | -0.682 |
| GI bleeding | -0.617 |
| GI surgery for neoplasm | -0.248 |
| Respiratory insufficiency after surgery | -0.140 |
| GI perforation/obstruction | 0.060 |
| Sepsis | 0.113 |
| Postarrest | 0.393 |
| If not in one of the specific groups above, then which major vital organ system was the principal reason for admission? | |
| Neurologic | -1.150 |
| Cardiovascular failure or insufficiency from | -0.797 |
| Respiratory | -0.610 |
| Gastrointestinal | -0.613 |
| Metabolic/renal | -0.196 |

Table S1: Coefficients for estimating the predicted mortality. ICH; intracranial hemorrhage, SDH; subdural hemorrhage, SAH; subarachnoid hemorrhage, GI; gastrointestinal.

Reference

1. Knaus WA, Draper EA, Wagner DP, Zimmerman JE. APACHE II: a severity of disease classification system. Crit Care Med. 1985 Oct;13(10):818–29.
